# PACAP for Retinal Health: Model for Cellular Aging and Rescue

**DOI:** 10.3390/ijms22010444

**Published:** 2021-01-05

**Authors:** Etelka Pöstyéni, Andrea Kovács-Valasek, Viktória Dénes, Adrienn Mester, György Sétáló, Róbert Gábriel

**Affiliations:** 1 Experimental Zoology and Neurobiology, University of Pécs, 7624 Pécs, Hungary; etelka91@gamma.ttk.pte.hu (E.P.); valasek@gamma.ttk.pte.hu (A.K.-V.); vdenes@gamma.ttk.pte.hu (V.D.); mesteradri@gmail.com (A.M.); 2 Department of Medical Biology, Medical School, University of Pécs, 7624 Pécs, Hungary; gyorgy.setalo.jr@aok.pte.hu; 3 János Szenthágotai Research Centre, 7624 Pécs, Hungary

**Keywords:** PACAP, somatostatin, retina, aging, cell density, peptidergic pathways

## Abstract

Retinal aging is the result of accumulating molecular and cellular damage with a manifest decline in visual functions. Somatostatin (SST) and pituitary adenylate cyclase-activating polypeptide (PACAP) have been implicated in neuroprotection through regulating disparate aspects of neuronal activity (survival, proliferation and renewal). The aim of the present study was to validate a transgenic model for SST-expressing amacrine cells and to investigate the chronic effect of PACAP on the aging of SSTergic and dopaminergic cells of the retina. SST-tdTomato transgenic mice that were 6, 12 and 18 months old were treated intravitreally with 100 pmol of PACAP every 3 months. The density of SST and dopaminergic amacrine cells was assessed in whole-mounted retinas. Cells displaying the transgenic red fluorescence were identified as SST-immunopositive amacrine cells. By comparing the three age groups. PACAP treatment was shown to induce a moderate elevation of cell densities in both the SST and dopaminergic cell populations in the 12- and 18-month-old animals. By contrast, the control untreated and saline-treated retinas showed a minor cell loss. In conclusion, we report a reliable transgenic model for examining SSTergic amacrine cells. The fundamental novelty of this study is that PACAP could increase the cell density in matured retinal tissue, anticipating new therapeutic potential in age-related pathological processes.

## 1. Introduction

Aging is the result of the accumulation of a wide variety of molecular and cellular damage over time [1], sharing common hallmarks with a range of diabetic as well as ischemic complications. Cardiovascular disease, inflammation and oxidative stress are major pathophysiological links among them [2,3].

Neuropeptides have an essential role in retinal physiology, as they affect information processing through multiple retinal peptidergic pathways [4,5,6]. The neuropeptides somatostatin (SST) and pituitary adenylate cyclase-activating polypeptide (PACAP) have various effects in many retinal physiological and pathological conditions [5,7,8,9]. These two neuromodulators share similarities in their functional relevance and structural parameters. Both peptides were isolated from hypothalamic tissue in the twentieth century and have two biologically active isoforms (SST—SST-14 and SST-28; PACAP—PACAP1-38 and PACAP1-27). Their pleiotropic cellular effects are exerted by widely distributed heptahelical transmembrane G-protein-coupled receptors (SST—SST1, SST2A, SST2B, SST3, SST4 and SST5; PACAP—PAC1-R, VPAC1-R and VPAC2-R) in the central and peripheral nervous system [10,11,12,13,14,15,16,17,18]. In the mouse retina, sparse SST immunoreactive cells have been reported as amacrine cells in the inner nuclear layer (INL) and displaced amacrine cells in the ganglion cell layer (GCL) [9], while cell bodies in the GCL, in addition to some amacrine cells and horizontal cells, show PACAP immunopositivity [19]. These two peptides were also studied in connection to a number of retinal pathologies such as the aging process and in diabetic retinopathy. PACAP treatments have exerted morphological, physiological and neurochemical protection in several disease models [20,21,22,23]. In an experimental model of diabetes, the synthetic somatostatin analogue octreotide (OCT) and PACAP exerted their neuroprotective effects by inhibiting vascular endothelial growth factor (VEGF) expression and retinal cell apoptosis [24]. In streptozotocin-induced diabetic retinopathy, the intravitreal injection of PACAP ameliorated structural changes in the retina (by affecting normal dopaminergic amacrine cell numbers compared to the untreated group) [21]. In the vitreous bodies of patients with diabetic retinopathy, significantly lower concentrations of SST were reported than in the controls [25]. A somatostatin deficit has been proven to trigger apoptosis and glial activation during diabetic retinopathy [26,27]. Some aspects of the diabetic complications show similarities with the symptoms of aging [28]. Age-related retinal functional and structural changes in the retina are well described in the aging human population worldwide. Neuropeptides and/or their analogs could offer an opportunity for treatments in certain retinal pathological conditions [7,29]. Previous studies have described that endogenous PACAP deficiency accelerated age-related retinal degeneration [22]. On the other hand, in the pathophysiology of age-related macular degeneration, OCT treatment stabilized visual acuity [30]. Ischemia is a major cause of visual impairments and is involved in the pathogenesis of many retinal diseases, such as diabetic retinopathy and age-related macular degeneration [31]. The anti-ischemic potential of both PACAP and OCT has been described in various disease models. Metabolomics analysis showed their protective effects in ischemia, where both PACAP and OCT ameliorated ischemia-induced oxidative stress, decreased cell death and downregulated VEGF overexpression [29]. With these facts in mind, these neuropeptides should be taken into account as therapeutic agents for decelerating age-related visual impairments [7,29]. Although our previous study described accelerated age-related retinal degeneration in PACAP-KO mice [22], and OCT treatment was demonstrated to stabilize visual acuity in age-related macular degeneration [30], the paucity of information on the relation between PACAP and retinal aging is striking.

In the present study, we aimed to investigate the chronic effect of PACAP treatment in the aging retina with particular regard to SST-expressing retinal cells. We found that multiple PACAP injections caused increased cell densities in the SSTergic amacrine cell population, suggesting synergistic regulation between these neuropeptides.

## 2. Results

### 2.1. Validation of SST-tdTomato Mouse Retina Engineered to Detect SST-Expressing Cells

**Figure 1:**
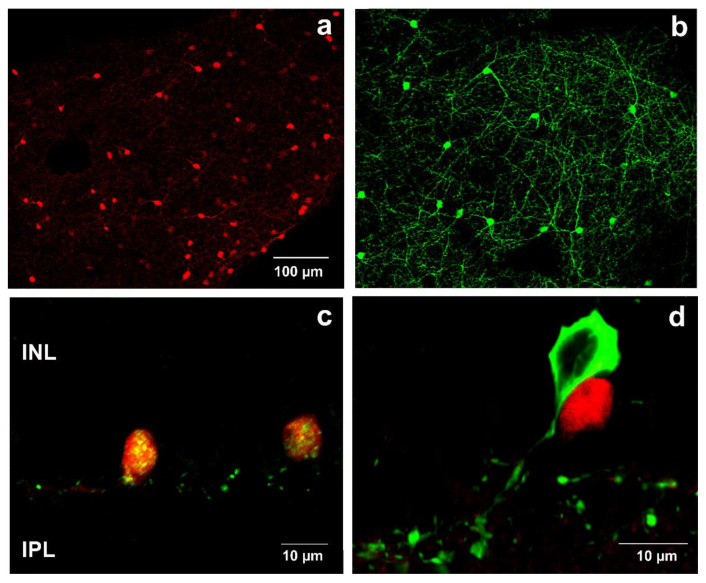
SST and TH immunohistochemistry in retinal whole mounts and sections in 6-month-old mouse retina. tdTomato-expressing cells (**a**) and TH-immunopositive neurons (**b**) in retinal whole mount. Co-localization of anti-SST antibody (labeled with AF 488-green) and red autofluorescent cells appeared as yellow in the merged image (**c**). Co-localization was not observed between SST cells (red) and TH immunopositive cells (green) (**d**). INL—Inner nuclear layer; IPL—Inner plexiform layer. Scale bar in (**a**) is valid for (**b**).

In Figure 1a, red fluorescent protein-expressing cells are shown in the whole-mounted control retina. Their pattern seemed to display a very similar distribution to that of the tyrosine hydroxylase (TH)-expressing dopaminergic amacrine cells (Figure 1b). To confirm the specificity for SST-expressing cells, SST immunostaining was performed. Doubled labeling showed that the red transgenic fluorescent cell population and green immunohistochemistry-driven fluorescent signal overlapped (Figure 1c). Furthermore, co-labeling with anti-TH antibody revealed that the red fluorescent protein-expressing and TH-immunopositive cell populations were entirely distinct (Figure 1d).

### 2.2. Quantitative Analysis: PACAP Treatment Caused an Increase in Cell Density

**Figure 2:**
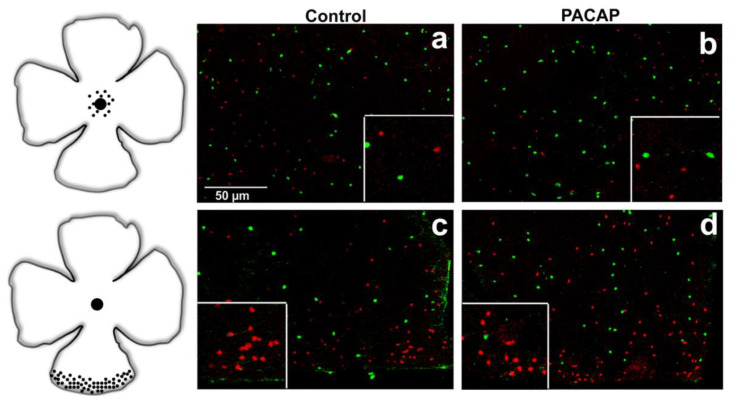
Distributions of SST (red) and TH (green) cells are shown in central (**a**,**b**) and peripheral (**c**,**d**) retina of 6-month-old mice as well as in saline-treated (**a**,**c**) and PACAP-treated (**b**,**d**) whole mounts. Scale bar is identical for all pictures.

**Figure 3:**
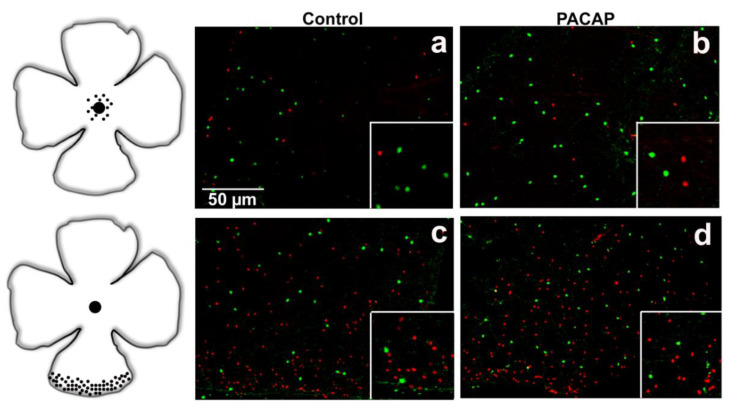
Distributions of SST (red) and TH (green) cells are shown in central (**a**,**b**) and peripheral (**c**,**d**) retina of 12-month-old mice. An increase in the density of both cell populations is clearly seen in the PACAP-treated (**b**,**d**) whole mount compared to saline-treated (**a**,**c**) retina. Scale bar is identical for all pictures.

**Figure 4:**
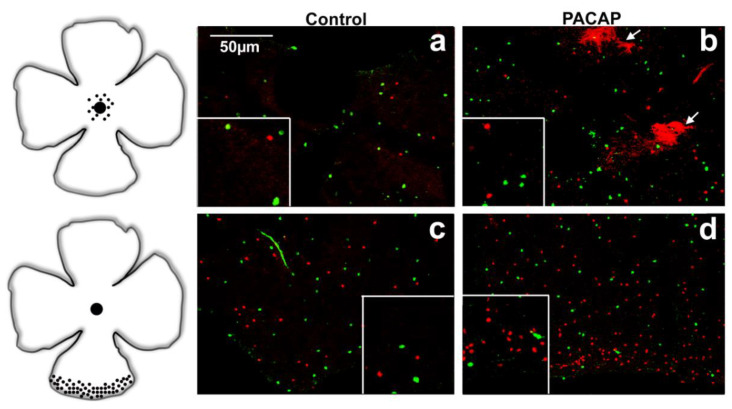
Distributions of SST (red) and TH (green) cells are shown in central (**a**,**b**) and peripheral (**c**,**d**) retina of 18-month-old mice. The increase in the SST and TH cell density is even more enhanced in the PACAP-treated (**b**,**d**) whole mount compared to saline-treated (**a**,**c**) retina. The arrows point to tdTomato-expressing glia-like cells. Scale bar is identical for all pictures.

In accordance with the available literature, a higher SST cell density was observed in the peripheral regions than in the central retina in the control tissues. At the same time, TH-positive cells displayed an evenly distributed pattern over the whole retina in all the examined groups (Figure 2, Figure 3 and Figure 4) (TH—[32,33]; SST—[9,34]). Besides the somatostatin-containing amacrine cell population, a few glia-like cells appeared in the oldest age group of the treated animals.

**Figure 5:**
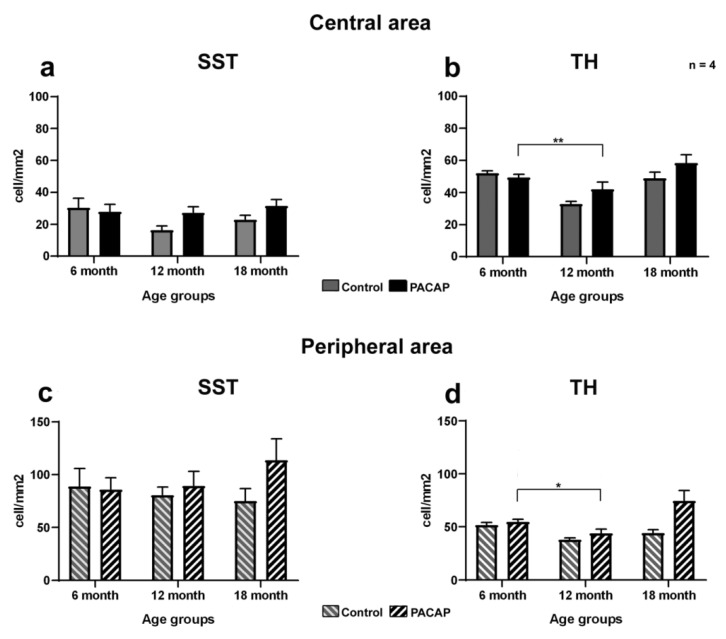
The SST (**a**,**c**)- and TH (**b**,**d**)-positive cell densities in central (**a**,**b**) and peripheral retinas (**c**,**d**) in different groups. Data presented as mean ± SEM, where * means *p* < 0.05 and ** means *p* < 0.01, compared to the cell densities of the 6-month-old PACAP-treated group.

Three experimental groups were designed to examine the long-term effects of PACAP injection on cell density: non-treated, saline-treated and PACAP-treated groups. The results of the quantitative analysis are presented in Table 1 and Figure 5.

To the best of our knowledge, multiple intravitreal injections caused no harm to the animals, as no significant difference was found between the non-injected and saline-injected retinas. Therefore, we opted to use the latter group as a control (sham manipulated). In the control retinas, neither the SST- nor the TH-positive cells displayed a statistically significant difference in central retinal cell density as the aging process progressed. In regard to PACAP treatments, a single injection caused no changes in SST or dopaminergic cell densities in the 6-month-old retinas (Figure 5a–d). In the 12-month-old group, a slight increase was observed in both cell populations. By contrast, in the 18-month-old retinas, which received PACAP treatment five times, a noticeable elevation was found, but with less statistical power. Notably, the peripheral area was rather strongly affected by PACAP, where an approximately 1.5-fold and a 1.7-fold increase were detected (Figure 5c,d), while a 1.37-fold and a 1.2-fold relative change were observed in the central retinal region for the SST and TH cell density, respectively.

## 3. Discussion

With time, the accumulation of age-related changes leads to progressively impaired function in the aging retina. The natural biological process of aging is responsible for neuronal cell loss in the retinal tissue [35]. The neuromodulator functions of PACAP and SST in the retina have been described in numerous mammalian species, such as the rat (SST—[36,37,38]; PACAP—[19]), humans (SST—[39]; PACAP—[40]) and the mouse (SST—[9]; PACAP—[41]). Due to their retinal presence under physiological circumstances, neuropeptide-based therapeutic strategies have been extensively researched for numerous vision-threatening diseases.

Very few studies have investigated the role of PACAP in the aging process, however, despite the accumulating evidence for its neuroprotective potential. PACAP-knockout mice have been reported to develop pre-senile amyloidosis and impaired articular cartilage formation [42,43]. In PACAP-knockout retinas, altered dendritic sprouting has been described [22]. Our results represent a novel, unique approach in demonstrating that intravitreal PACAP treatment has a beneficial long-lasting effect on age-related neuronal cell loss.

The retinal area generally increases with age, and this enlargement could lead to a reduction in cell density [44,45]. At the 12-month time point, the non-treated and saline-injected controls showed small, statistically insignificant losses in SST-positive cell densities. At the same time, in the PACAP-treated retinas, we found normal cell densities in central areas and, simultaneously, increased cell densities at the periphery. In the oldest treated groups, both cell types showed higher cell densities in both central and peripheral retinal areas, while the cell densities in the non-treated group decreased. In both groups, we noticed glia-like cells in the central area, which were present in higher numbers in the PACAP-treated retinas. The glial cells could, in theory, contribute to neuroprotection and help to maintain tissue homeostasis [46,47]. The increased glial cell activation could also be a specific reaction to the homeostatic disturbances occurring during the aging process. Their upregulation by PACAP may promote higher neuronal cell densities in these groups. PACAP receptor expression was reported on various immune cell populations representing distinct patterns. The observation suggests a link between the appearance of glia-like cells and the repeated PACAP injections [48].

One possible explanation for the increased cell numbers is the promotion of neuronal stem cell activation and/or differentiation. In the adult retina, a small population of proliferating stem cells resides in the marginal zone of the ciliary body [49]. Although normally resting, they can be activated by exogenous growth factors or injuries [50,51]. Our findings suggest that PACAP might be a candidate for stimulating retinal stem/progenitor cells, opening a new avenue for future studies. Another prominent feature of PACAP-mediated processes that compensates for cell loss is the well-known anti-apoptotic effect. It was described in cortical neuronal cultures, where it relies on the activation of cAMP response element-binding protein (CREB)-mediated gene expression and action potential firing [52]. The PACAP-induced phosphorylation effect on CREB has also been described in the retina [53]. The CREB family controls neuronal survival in many neuronal subtypes through the regulation of the transcription of certain survival factors (such as Bcl-2), while the disruption of CREB has been shown to result in neurodegenerative processes in the central nervous system [54,55,56].

Furthermore, our findings raise the possibility that the effects of PACAP and SST are cumulative. Speculatively, this may have significant clinical implications in the future. The potency of these peptides is based on their receptor signaling through multiple cellular pathways. The underlying molecular processes of SST- and PACAP-receptor signaling include the inhibition of proapoptotic molecules (e.g., caspase-3, caspase-8, FasL and calpain-2), meaning that they could be very well suited for reducing the harmful effects of diabetic retinopathy [21,57,58,59]. SST eyedrops have helped in the prevention of neurodegeneration and b-wave abnormalities in streptozotocin-induced diabetic retinopathy [58]. In optic nerve crush-induced retinal ganglion cell apoptosis, PACAP treatment upregulates Bcl-2 expression and inhibits caspase-3 expression [53]. Intraocular PACAP injection attenuates proapoptotic signals (p-p38MAPK and caspase-3, -8 and -12) and promotes anti-apoptotic factors (p-Akt, p-ERK1, p-ERK2, PKC and Bcl-2) in experimental diabetic retinopathy [21]. The signal transduction pathways activated by SST and PACAP are convergent, and share identical members, which raises the possibility that they may enhance each other. The long-lasting neuroprotective effects of PACAP on SST-containing neuronal cells support the potential therapeutic effectiveness of neuropeptides in counteracting the aging process. The results highlight the importance of further understanding corroborative neuropeptide pathway mechanisms, and our present study represents a significant step in that direction.

## 4. Materials and Methods

### 4.1. Animals and PACAP Treatment

Experiments were performed on 6-, 12- and 18-month-old C57Bl/6J TdTomato transgenic mice (n = 4 in each age group and condition) that were purchased from the Institute of Experimental Medicine of the Hungarian Academy of Sciences. The mice were generated by crossing the homozygote SST/iresFlpo (Tm3) mouse line with homozygote GT(ROSA)26Sor_CAG/FSF_TdTomato animals. These transgenic mice express enhanced red fluorescent protein (TdTomato) in somatostatin-containing cells. The construct was validated by PCR tests. The animals were housed under identical conditions: with water and food ad libitum, in a temperature- and light-controlled room (12/12 h light/dark cycles, 23 °C). The animal housing and experimental procedures were reviewed and approved by the ethics committee of the University of Pécs (BAI/35/51-58/2016). Under isoflurane anesthesia, 1.5 µL of 0.3 µg/µL (100 pmol) PACAP1-38 (Bio Basic Canada Inc., Markham, ON, Canada) was injected intravitreally with a Hamilton syringe (a 10 µL microsyringe). The left eye served as a control, and it received the same volume of vehicle treatment (0.9% saline solution). The intervals between the treatments were 3 months, which means that the numbers of injections varied with age group: one for the 6-month group, three for the 12-month group and five for the 18-month group.

### 4.2. Immunohistochemistry on Retinal Whole Mounts

After animals were sacrificed by an overdose of isoflurane anesthetic, the eyes were immediately dissected. The retinas were fixed in freshly prepared 4% PFA for 2 h at room temperature. The samples were rinsed in phosphate buffered saline (PBS) and then incubated in 1%TritonX-ABS for an hour. The retinas were incubated with anti-TH antibody (rabbit, 1:1000; Abcam, Cambridge, UK) for 72 h at 4 °C in an incubator–rotator (Enviro Genie, Bohemia, NY, USA). After intensive washes with 1% Triton-PBS, the retinas were incubated with the secondary antibody (goat-anti-rabbit, AlexaFluor-488, 1:1000; ThermoFisher Scientific, Budapest, Hungary) overnight at 4 °C in the incubator–rotator. Samples were washed with 1% TritonX-PBS and mounted with Fluoromount-G (ThermoFisher Scientific, Budapest, Hungary). Photographs were taken with an Olympus Fluorview FV-1000 Laser Confocal Scanning Microscope (Olympus, Tokyo, Japan). The density (cells/mm^2^) of SST autofluorescent and TH-positive cells was assessed in the central and peripheral areas. Quantitative data were obtained using an image analysis program (Fiji is just Image J 1.52p, NIH Bethesda, MD, USA). The values of the cell densities are expressed as means ± SEM. Statistical comparisons were made using nonparametric Mann–Whitney analysis.

### 4.3. Immunohistochemistry on Retinal Sections

Eyecups were fixed in 4% PFA for 2 h at room temperature. The specimens were cryoprotected in 15% and 30% sucrose solutions at 4 °C. Cryo-sections were incubated with anti-STT (rabbit, 1:1000; BMA Biomedicals, Augst, Switzerland) and anti-TH (rabbit, 1:1000; Abcam, Cambridge, UK) antibodies overnight, followed by the corresponding secondary fluorescent antibodies (goat-anti-rabbit, AlexaFluor-488; Life Technologies, ThermoFisher Scientific, Budapest, Hungary; 1:1000) for 2 h. For the controls, we omitted the primary antibodies from the incubation steps, which resulted in non-staining. Photographs were taken with an Olympus Fluorview FV-1000 Laser Confocal Scanning Microscope (Olympus, Tokyo, Japan), using the same settings for one marker.

## Figures and Tables

**Table 1:** Summary of SST and TH cell densities in central and peripheral areas of 6- 12- and 18-month-old retinas. Values are expressed as means ± SEM (n = 4).

	SST Cell Density (Cell/mm^2^)					
	Central Retina			Peripheral Retina		
	6 m	12 m	18 m	6 m	12 m	18 m
Non-treated group	46.0 ± 12.3	22.5 ± 5.2	27.8 ± 6.1	64.0 ± 12.5	71.2 ± 6.8	64.9 ± 16.9
Saline-treated group	30.4 ± 6.0	16.3 ± 2.7	22.9 ± 2.7	88.8 ± 17.0	80.7 ± 7.5	75.1 ± 11.9
PACAP-treated group	27.9 ± 4.5	27.3 ± 3.7	31.5 ± 3.9	85.9 ± 11.3	89.5 ± 13.5	113.6 ± 20.3
	**TH Cell Density (Cell/mm^2^)**					
	**Central Retina**			**Peripheral Retina**		
	**6 m**	**12 m**	**18 m**	**6 m**	**12 m**	**18 m**
Non-treated group	50.1 ± 5.2	35.4 ± 2.2	43.5 ± 4.2	41.2 ± 4.8	37.7 ± 1.9	43.8 ± 3.2
Saline-treated group	52.1 ± 1.5	32.9 ± 1.5	49.0 ± 3.7	51.8 ± 2.3	37.9 ± 1.7	44.3 ± 3.1
PACAP-treated group	49.4 ± 1.9	42.1 ± 4.5	58.4 ± 5.1	54.9 ± 2.4	43.9 ± 3.9	74.7 ± 9.4

## Data Availability

Original raw data are available from authors on request.

## Abbreviations

SST	somatostatin
PACAP	pituitary adenylate cyclase-activating polypeptide
INL	inner nuclear layer
GCL	ganglion cell layer
VEGF	vascular endothelial growth factor
IPL	inner plexiform layer
OCT	octreotide
TH	tyrosine hydroxylase
cAMP	cyclic adenosine monophosphate
CREB	cAMP response element-binding protein
p38 MAPK	p38 mitogen-activated protein kinases
ERK	extracellular-signal-regulated kinase
PKC	protein kinase C
